# Astrocyte Senescence and Alzheimer’s Disease: A Review

**DOI:** 10.3389/fnagi.2020.00148

**Published:** 2020-06-09

**Authors:** Xiaojuan Han, Tianying Zhang, Huanhuan Liu, Yajing Mi, Xingchun Gou

**Affiliations:** Shaanxi Key Laboratory of Brain Disorders & Institute of Basic and Translational Medicine, Xi’an Medical University, Xi’an, China

**Keywords:** Alzheimer’s disease, astrocytes, senescence, senescence-associated secretory phenotype, senolytic drugs, tau aggregate

## Abstract

Astrocytes are the largest group of glial cells in the brain and participate in several essential functions of the central nervous system (CNS). Disruption of their normal physiological function can lead to metabolism disequilibrium and the pathology of CNS. As an important mechanism of aging, cellular senescence has been considered as a primary inducing factor of age-associated neurodegenerative disorders. Senescent astrocytes showed decreased normal physiological function and increased secretion of senescence-associated secretory phenotype (SASP) factors, which contribute to Aβ accumulation, tau hyperphosphorylation, and the deposition of neurofibrillary tangles (NFTs) in Alzheimer’s disease (AD). Astrocyte senescence also leads to a number of detrimental effects, including induced glutamate excitotoxicity, impaired synaptic plasticity, neural stem cell loss, and blood–brain barrier (BBB) dysfunction. In this review article, we have summarized the growing findings regarding astrocyte senescence and its putative role in the pathologic progress of AD. Additionally, we also focus on the significance of targeting astrocyte senescence as a novel and feasible therapeutic approach for AD.

## Introduction

Alzheimer’s disease (AD) is a chronic degenerative disorder of the brain related to progressive decline of memory and cognition (McGeer and McGeer, 2007; Long and Holtzman, 2019). The disease is characterized by brain atrophy, extracellular accumulation of beta-amyloid peptide (Aβ) (Glenner and Wong, 1984; Hsiao et al., 1996), neurofibrillary tangles (NFTs) composed of hyperphosphorylated tau protein (Goedert et al., 2006), and loss of synapses and dysfunctions of neurotransmission (DeKosky and Scheff, 1990; Rajmohan and Reddy, 2017), as well as neuroinflammation (Heneka et al., 2015).

Many of the cellular pathologies of AD present on neurons, such as neuronal extracellular deposits of Aβ, intracellular deposition of neurofibrillary tangles (NFTs), and Lewy bodies (Hansen et al., 1990; Hardy and Higgins, 1992; Goedert et al., 2006). These classical pathologies are still central to diagnosing AD. However, although neurons have significant correlations with AD, other cell types and factors in the brain may also contribute to cognitive decline during AD. Additionally, astrocytes are the major glial cells and are vital for the normal physiological functions of the central nervous system (CNS) (Sofroniew and Vinters, 2010; Matias et al., 2019). They perform critical roles in regulation of homeostasis and metabolism of the neurons, mediating uptake and recycling of neurotransmitters. Astrocytes also play a key role in maintenance of the blood–brain barrier (BBB). They also act as modulators of synaptic plasticity and transmission, supporting the view that astrocytes play an integral role in the initiation and progression of cognitive decline and AD (Acosta et al., 2017; Palmer and Ousman, 2018; Munger et al., 2019).

Aging is considered the most significant risk factor for the occurrence and development of AD (Kritsilis et al., 2018). The incidence of AD has been shown to increase with advancing age and cellular senescence (Baker et al., 2011). Studies regarding to the link and role of senescence in age-related diseases have become increasingly common, and are gradually becoming a new research area (Bhat et al., 2012; Baker and Petersen, 2018). Transcriptome analysis of AD and the aged human brain showed neurons and other non-neuronal CNS cell types including astrocytes, microglia, and oligodendrocytes displayed senescence-associated phenotypes (Salminen et al., 2011; Bhat et al., 2012; Boccardi et al., 2015; Boisvert et al., 2018; Bussian et al., 2018; Zhang P. et al., 2019). Although studies regarding astrocyte senescence during AD and the effects of senescent astrocytes in AD progression have recently increased, an understanding of its precise mechanisms is still lacking. Here, we review our current view on the role of astrocyte senescence in the pathogenesis of AD.

## Astrocytes

Astrocytes are the most abundant type of glial cells in the brain and are classified into two main groups: fibrous astrocytes and protoplasmic astrocytes (Sofroniew and Vinters, 2010). Fibrous astrocytes are characterized by the presence of numerous fibrils in their cytoplasm and are distributed mainly in the white matter. Protoplasmic astrocytes are typically located in the gray matter and have thick, short, highly branched processes with fewer fibrils (Sofroniew and Vinters, 2010). Despite heterogeneity of astrocytic subtype, these glia are responsible for multifarious complex and essential functions for CNS physiology, including the provision of nutrients to the neuron, regulation of synaptic plasticity, releasing transmitters (called gliatransmitters) in a Ca^2+^-dependent manner, supporting the blood–brain barrier (BBB), and maintaining the extracellular ion balance (Hussaini and Jang, 2018; Verkhratsky and Nedergaard, 2018).

Astrocytes have been shown to become activated in response to various stimuli and diseases of the CNS. One common and classical feature of reactive astrocytes is the release of a variety of effector molecules including chemokines, cytokines, and proteases (Liddelow et al., 2017; Ahmad et al., 2019). Interestingly, these factors overlap with the secretions of senescent astrocytes (Boisvert et al., 2018). The proteins associated with astrocyte activation, glial fibrillary acidic protein (GFAP) and vimentin, increased during aging (Porchet et al., 2003). Also, senescent astrocytes share many of the similar phenotypes with A1-like reactive astrocytes, including cellular morphological change and proinflammation secretions. It is possible that many previous studies, which focused on reactive astrocytes, may have been focusing on senescent astrocytes. Although Cohen et al. reviewed the different features between reactive and senescent astrocytes, it is necessary to elucidate the characteristics of astrocyte senescence further (Cohen and Torres, 2019). In the next section, we will describe the phenotypes of senescent astrocytes in more detail.

## Astrocyte Senescence: Astrosenescence

According to Cohen et al., astrocytes can initiate a senescence program similar to that of other cell types in response to various stressors, termed “astrosenescence” (Cohen and Torres, 2019). Ponten and Macintyre (1968) compared the characteristics of glial cells isolated from malignant and benign tissue to examine the effect of long-term culturing on CNS cells. This study indicated that primary cells from normal tissue had a limited lifetime and stopped dividing after a set number of passages. This might be the first study to examine replicative senescence in glia cells. With time, numerous studies demonstrated that after exhausted replication, oxidative stress, proteasome inhibition, high glucose, or HIV infection, astrocytes show changes in several classical hallmarks of cellular senescence.

Primary astrocytes isolated from the cerebral cortex that underwent replicative senescence displayed a series of established markers of cellular senescence including a growth arrest, increased expression of senescence-associated genes *p53* and *p21*^*WAF1*^, and increased senescence-associated β-galactosidase (SA-β-Gal) activity (Evans et al., 2003; Blasko et al., 2004; Pertusa et al., 2007). Astrocytes have shown to undergo stress-induced premature senescence as well. For example, after ionizing radiation or H_2_O_2_ or proteasome inhibitor treatment, both human and mouse astrocytes displayed classical senescence features, such as decreased proliferation, increased SA-β-Gal activity, and the upexpression of *p53*, *p21*^*WAF1*^, and *p16*^*INK4A*^ (Bitto et al., 2010; Turnquist et al., 2019). Notably, astrocytes were found to be more sensitive to senescence-inducing stimuli than fibroblasts (Gorg et al., 2015, 2018). Also, Aβ oligomers can induce cellular senescence and promote production of senescence-associated secretory phenotypes (SASPs) in human astrocytes (Mombach et al., 2015). Furthermore, human astrocytes infected with HIV showed signs of DNA damage and premature senescence (Cohen et al., 2017). Therefore, astrocytes have been shown to undergo cellular senescence *in vitro* and *in vivo* due to various stimuli and factors.

## Characteristics of Astrocyte Senescence

**FIGURE 1:**
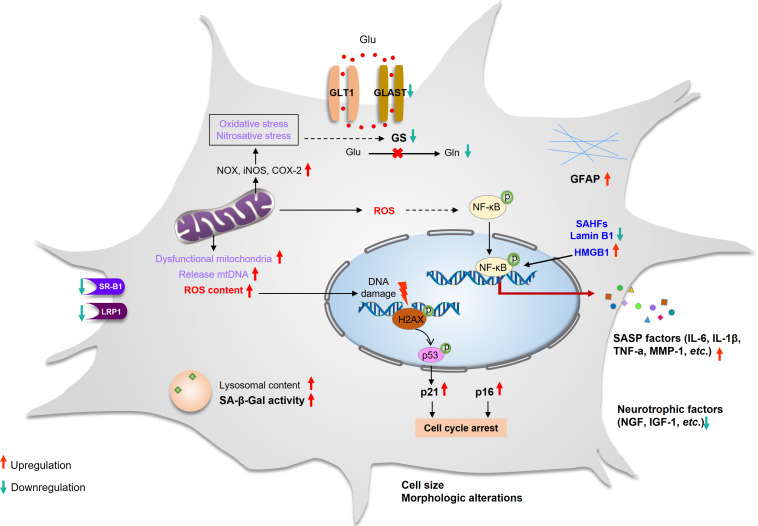
Characteristics of astrocyte senescence. Senescent astrocytes that have undergone cell cycle arrest and have enlarged sharply. The expression of GFAP increased, whereas the expression of glutamate transporters (GLAST and GS) and SR-B1 and LRP1 decreased. The lysosomal content has increased, and the lysosomes have high β-galactosidase activity and dysfunctional mitochondria that produce high levels of ROS and release mtDNA. They have DNA damage and SAHFs, and their nuclear integrity is compromised due to the loss of laminB1. The elevated ROS activates the NF-κB pathway and promotes SASP production. The secretion of neurotrophic factors is decreased. GFAP, glial fibrillary acidic protein; GLAST, glutamate aspartate transporter; GLT-1, glutamate transporter-1; GS, glutamine synthetase; iNOS, inducible nitric oxide synthase; LRP1, lipoprotein receptor–related protein 1; NOX, NADPH oxidase; ROS, reactive oxygen species; SAHFs, senescence-associated heterochromatic foci; SASP, senescence-associated secretory phenotype; SR-B1, scavenger receptor B1.

Cellular senescence is a catchall for a set of states in which cells stop dividing and then exhibit a multitude of cellular and molecular changes. Evidence suggests that there is a significant variation in the senescent phenotype that is dependent on both cell type and triggering insults (Gorgoulis et al., 2019). Senescent astrocytes exhibit both classic characteristics as well as other cell types and also demonstrated particular phenotypes (Bhat et al., 2012; Buhlman, 2017; Baker and Petersen, 2018). Key features of senescent astrocytes include the following: permanent cell cycle arrest, altered morphology, increased GFAP and vimentin, chromatin alterations and formation of senescence-associated heterochromatic foci (SAHFs), upexpression of high-mobility group B (HMGB) proteins, reduced expression of nuclear lamina protein laminB1, downregulation of neurotrophic growth factors, and upregulation of SASP factors as well as SA-β-Gal (Bitto et al., 2010; Boccardi et al., 2015; Chinta et al., 2015; Boisvert et al., 2018; Turnquist et al., 2019; Figure 1).

### Cell Arrest and GFAP

Typically, senescent astrocytes exhibit permanent cell cycle arrest as well as other cell types, which is thought to be regulated by the p53/p21^WAF1^ and p16^INK4A^/pRB pathway (Evans et al., 2003; Bitto et al., 2010; Turnquist et al., 2019). p21^*WAF1*^ is namely the CIP/KIP (CDK interacting protein/kinase inhibitory protein) that is capable of inhibiting CDK2, but paradoxically, it is also necessary for cell cycle progression (Hernandez-Segura et al., 2018). In the context of astrocyte senescence, p53 upregulates the expression of p21^WAF1^, which inhibits cyclin D–dependent kinase CDK2 activity and the initial cell cycle arrest. Interestingly, this p53-dependent stable proliferative arrest was independent of telomere erosion in human astrocyte *in vitro* study (Evans et al., 2003). p16^INK4A^ is a member of the INK4A family that mediates permanent cell cycle arrest by inhibiting CDK4 and CDK6, which leads to retinoblastoma protein (RB) hypophosphorylation, blocking cell cycle entry to the S phase (Hernandez-Segura et al., 2018). Importantly, as its expression increased in the brain with time, p16^INK4A^ is also a biomarker of natural brain aging (Berchtold et al., 2008; Baker and Petersen, 2018).

Additionally, GFAP is a class III intermediate filament protein, which is the most widely used marker for astrocytes (Eun et al., 2016). In the human brain, the level of GFAP was significantly increased in the hippocampus in people over 65 years of age. GFAP upexpression has been the general change observed in astrocyte senescence *in vitro* and *in vivo* (Nichols et al., 1993; Boisvert et al., 2018; Lye et al., 2019). Larsson et al. have demonstrated that cell proliferation in the granular layer of the dentate gyrus is increased after knockout GFAP/vimentin (Larsson et al., 2004). Several studies have demonstrated that the expression of GFAP appears to increase with aging in rodents and human astrocytes (Iram et al., 2016), although activated astrocytes also involve the upregulation expression of GFAP (Liddelow et al., 2017). This implies that astrocyte senescence associated with an increase in GFAP expression, which results in the upregulation of GFAP, may be a new biomarker of astrocyte senescence (Figure 1).

### Nuclear Changes

In senescent cells, chromatin alterations and remodeling including formation of SAHFs and nuclear DNA enriched for histones modification were stained densely by DAPI (Kosar et al., 2011). These nuclear changes have been associated with attenuated expression of proliferation-promoting genes, which leads to irreversible cell cycle arrest of senescence (Hernandez-Segura et al., 2018; Gorgoulis et al., 2019). Unlike γH2AX, which is a ubiquitously expressed DNA damage response (DDR) marker, the formation of SAHFs in senescent cells varies between cell types (Seoane et al., 2017; Gorgoulis et al., 2019). Studies have shown that cultured human astrocytes displayed both increased levels of γH2AX and formation of SAHFs (Myung et al., 2008; Bitto et al., 2010; Souza et al., 2015; Seoane et al., 2017; Figure 1). Senescent astrocytes showed nuclear enlargement, which is generally present in specific cell types such as fibroblasts and CNS cells (Yoon et al., 2016; Bang et al., 2019). When DNA damage and DNA repair occur during the cell cycle, the size of the cell and nucleus is slightly increased (Amodeo and Skotheim, 2016). It seems that the enlargement of the nucleus in senescent cells may be caused by DNA damage and cell cycle arrest.

Senescent astrocytes also display changes in nuclear morphology and integrity of the nuclear envelope owing to the downregulation of nuclear lamina proteins, such as lamin B1 (Freund et al., 2012). Additionally, HMGB1 plays a crucial role in DDR and cellular inflammation (Enokido et al., 2008; Castiglioni et al., 2011), which is increased in astrocytes with aging (Enokido et al., 2008; Figure 1).

### Senescence-Associated Secretory Phenotype

Oxidative stress-induced and cultured senescent astrocytes cause several transcriptomic changes. More specifically, genes associated with proinflammation cytokines, such as interleukin (IL)-6, IL-8, chemokines, and proteinases were upregulated (Crowe et al., 2016; Hou et al., 2018). These proinflammation factors are termed the SASPs, which are considered to be a downstream consequence of the DDR (Salminen et al., 2011; Seoane et al., 2017). Additionally, Aβ peptides and environmental toxins, such as ammonia and paraquat, have also been shown to induce senescence in cultured astrocytes with increased production of SASP. SASP is highly heterogeneous in senescent astrocytes that are induced by different stimuli (Bhat et al., 2012; Crowe et al., 2016; Clarke et al., 2018; Hou et al., 2018). However, the high expression of IL-6 is a more common feature in astrocyte senescence (Blasko et al., 2004; Hou et al., 2017).

Several studies indicated that SASP-related genes upregulated in senescent astrocytes is mediated by the p38/MAPK and NF-κB pathway (Chien et al., 2011; Salminen et al., 2011; Mombach et al., 2015). Bhat et al. reported that inhibition of p38/MAPK activity both in presenescent and senescent human astrocytes suppress the increased level of SASP factors (Bhat et al., 2012). Following the hypothesis that DNA damage is an essential driver of SASP, HMGB1 may be another important regulator. In the brain, HMGB1 is upexpressed in astrocytes during aging (Enokido et al., 2008). Davalos et al. reported that HMGB1 could increase NF-κB transactivation efficiency through interaction with NF-κB complexes and therefore augment and strengthen the inflammatory reaction of the SASP (Qiu et al., 2010; Davalos et al., 2013). Recently, the cyclic GMP-AMP synthase (cGAS)/ stimulator of interferon (IFN) genes (STING) pathway, NOTCH signaling, and mammalian target of rapamycin (mTOR) signaling have also been shown to regulate SASP in fibroblast and other cell types (Herranz et al., 2015; Capell et al., 2016; Hoare et al., 2016; Loo et al., 2019). However, the mechanisms involved in CNS cells have not been extensively studied. SASPs have been shown to operate as autocrine and paracrine signals to reinforce the senescent state and induce senescence or degenerative changes of the surrounding bystander cells (Coppe et al., 2010; Acosta et al., 2013). These observations indicate that SASPs can generate a low-level, chronic inflammation and an age-dependent detrimental cycle to strengthen the senescence state and enhance age-related neurodegenerative disorders.

### Lysosomal and Mitochondrial Dysfunction

The upregulation of lysosomal proteins and increased lysosomal content is the main characteristic of cellular senescence (Lee et al., 2006). Enhanced lysosomal content can be detected by measuring the activity of the lysosomal enzyme senescence-associated beta-galactosidase (SA-β-Gal), which is measured at pH 6.0 using in situ staining with the chromogenic substrate X-gal (Kurz et al., 2000; Lee et al., 2006). Therefore, SA-β-Gal is used as the most common hallmark for detecting senescent cells. Senescent astrocytes showed elevated SA-β-Gal activity *in vitro* and *in vivo* (Evans et al., 2003; Bitto et al., 2010; Bhat et al., 2012; Cohen and Torres, 2019). Consistent with others, our previous research also demonstrated the accumulation of dysfunctional lysosomes in stress-induced premature senescence (SIPS) (Han et al., 2016; Tai et al., 2017).

It is known that the number of mitochondria increases during senescence. However, the membrane potential of mitochondria is decreased, leading to intensified ROS production and the release of mitochondrial DNA (mtDNA) (Passos et al., 2007). Meanwhile, elevated mtDNA in the cytoplasm can lead to STING-meditated SASP production (Li and Chen, 2018). Enhanced mitochondria content during senescence could be the result of the accumulation of dysfunctional mitochondria and reduced mitochondrial fission (Tai et al., 2017; Kim et al., 2018). In addition, impaired mitophagy is also likely to contribute to dysfunctional mitochondria accumulation in senescent cells (Fivenson et al., 2017; Vasileiou et al., 2019). Studies using either senescent astrocytes from aged rats or *in vitro* culturing showed that mitochondrial dysfunction and damage was associated with an increase in oxidative/nitrosative stress, RNA oxidation, upregulation of ROS, and inducible nitric oxide synthase (iNOS) expression levels (Figure 1; Pertusa et al., 2007; Bellaver et al., 2017; Bang et al., 2019).

### Glutamate Signaling Dysfunction

Due to the high expression of glutamate transporters, GLAST (human homologs, EAAT1) and GLT-1 (human homologs, EAAT2) (Zhang X. et al., 2019), astrocytes can further regulate neuronal function via the efficient uptake of the synaptically released excitatory and inhibitory neurotransmitters glutamate and γ-aminobutyrate (GABA). Glutamate is the primary excitatory neurotransmitter in the mammalian CNS and is critical for learning and memory (Verkhratsky and Kirchhoff, 2007). Nevertheless, excessive extracellular glutamate levels lead to neuronal death as a result of glutamate excitotoxicity (Danbolt, 2001). The released glutamate from neurons is mainly taken up into astrocytes by GLAST and GLT-1 and is then converted to glutamine by glutamine synthase (GS), which is expressed in astrocytes (Pajarillo et al., 2019; Zhang X. et al., 2019). The expression and activity of GS are age dependent. The protein level of GS has been found to decrease dramatically in aged astrocytes (Bellaver et al., 2017; Shi et al., 2017). In addition, the expression of the glutamate transporter GLAST displays an age-dependent decrease. However, there was an increase in the expression of GLT-1 in senescent astrocytes (Bellaver et al., 2017). Other research has shown only reduced expression and activity of GS, with no changes observed in the expression of GLAST andGLT-1 in senescent astrocytes (Boisvert et al., 2018). Additionally, the activity of GS is very sensitive to nitrosative and oxidative stress (Knorpp et al., 2006; Bellaver et al., 2017). Therefore, oxidative stress could decrease the ability of astrocytes to supply metabolic substrates to neurons and aggravate the impairment of GS activity (Crowe et al., 2016; Gonzalez-Reyes et al., 2017). It is likely that senescent astrocytes with reduced capacity for glutamate uptake and clearance contribute to glutamate excitotoxicity in neurodegenerative diseases.

### Cholesterol Synthesis

Cholesterol is an essential metabolic substrate for the normal physiological functions of the brain, supporting neuronal homeostasis, synaptic integrity, and receptor function. In the CNS, cholesterol content is largely independent of dietary intake because of the existence of the BBB (Giudetti et al., 2016). Astrocytes are thought to be crucial in brain cholesterol synthesis and transport owing primarily to their expression of sterol regulatory element-binding protein 2 (SREBP2) and apoE (Ferris et al., 2017). SREBP2 is an important transcription factor that regulates the expression of HMG-CoA reductase (HMGCR), the rate-limiting enzyme of cholesterol synthesis (Kim et al., 2010). In senescent astrocytes, the expression of HMGCR and genes associated with cholesterol synthesis is significantly decreased, while the mRNA level of cholesterol transport-related genes is increased (Boisvert et al., 2018). It appears that there is an overall dysregulation of cholesterol metabolism in senescent astrocytes, and this may lead to decreased synaptic support.

## Functional Consequences of Senescent Astrocytes in Alzheimer’s Disease

Alzheimer’s disease (AD) is a progressive neurodegenerative disease causing cognitive, memory, and behavioral dysfunctions in older age. The pathophysiological mechanism of AD has been extensively studied for many years, which has led to several hypotheses, such as the Aβ, tau, cholinergic, and inflammation hypotheses (Hardy and Higgins, 1992; McGeer and McGeer, 2007; Hampel et al., 2018).

Numerous studies have shown that impaired astrocytes are involved in the initiation and progression of AD (Acosta et al., 2017; Gonzalez-Reyes et al., 2017; Matias et al., 2019). One most likely mechanism is astrocytic neuroinflammation (Benzing et al., 1999; Blasko et al., 2004). Also, a common feature of astrocyte senescence is the production of proinflammatory factors known as the SASP. In addition, studies have shown evidence that astrocytes surrounding Aβ plaques are positive for IL-6, a key SASP component (Bhat et al., 2012; Munger et al., 2019). All these data support the view that astrocyte senescence is a key and novel contributor to AD pathogenesis.

**FIGURE 2:**
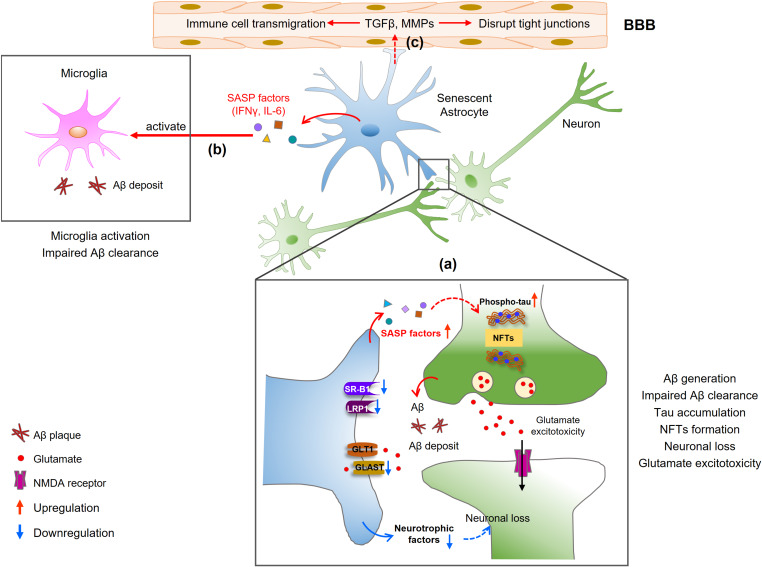
Mechanisms of senescent astrocytes in the pathologies of AD. **(a)** SASP factors produced by senescent astrocytes induce Aβ generation and tau hyperphosphorylation, contributing to Aβ deposition and NFTs formation. Neurons release glutamate, which cannot be cleared from the synapse by senescent astrocytes, leading to an accumulation of extracellular excitotoxic glutamate. The reduced secretion of neurotrophic growth factors (NGF, IGF-1) in senescent astrocytes may lead to decreased neuronal growth or increased neuronal loss. **(b)** Senescent astrocytes secrete IFNγ and IL-6, which activate microglia and limit Aβ uptake by microglia. **(c)** SASP factors also disrupt endothelial tight junctions and induce leukocyte transmigration, resulting in BBB disruption.

The first study to investigate the role of astrocyte senescence in AD involved human astrocytes treated with the Aβ_1__–__42_ oligomer *in vitro* (Bhat et al., 2012). After treatment, astrocytes displayed classical phenotypes of cellular senescence including increased SA-β-Gal activity and increased p16^*INK4A*^ expression. Most notably, not only was there an age-dependent increase in p16-positive astrocytes in the frontal cortex tissue, there was a further increase in senescent astrocytes in brain tissue from AD patients compared to non-diseased tissues from age-matched individuals (Bhat et al., 2012; Turnquist et al., 2016). Also, a recent study suggested that removal of senescent astrocytes and microglia by senolytic agents or genetic ablation prevents or inhibits NFTs formation and neurodegeneration in AD and tauopathy mouse models (Bussian et al., 2018; Mendelsohn and Larrick, 2018), suggesting that astrocyte senescence contributes to the pathogenesis of neurodegeneration in AD. In this part, we summarize the data that demonstrate the effects of senescent astrocytes in AD (Figure 2).

### Aβ Accumulation

Inefficient Aβ clearance is detrimental in AD. Many studies have shown that astrocytes play an important role in Aβ clearance and degradation (Nicoll and Weller, 2003; Liu et al., 2017). Astrocyte receptors involved in uptake and clearance of Aβ are the low-density lipoprotein receptor-related protein 1 (LRP1) and scavenger receptor B1 (SR-B1) (Basak et al., 2012; Ries and Sastre, 2016). However, the mechanisms governing the receptor-mediated uptake of Aβ are not fully understood. Notably, in aged astrocytes, the expressions of LRP1 and SR-B1 are reduced (Iram et al., 2016), suggesting the ability of senescent astrocytes to uptake and degrade Aβ may be impaired.

In AD, senescent astrocytes are observed in regions surrounding Aβ plaque and the appearance of SA-β-Gal–positive astrocytes was earlier than Aβ accumulation (Bhat et al., 2012). The expression of BACE1 and γ-secretase subunits PS1, PS2, and PEN2 is increased by cellular stress in astrocytes (Grolla et al., 2013; Frost and Li, 2017). Subsequent studies have demonstrated that Aβ_1–42_ itself can induce astrocyte senescence and further induce the astrocytic APP and β-secretase processing, resulting in a further increase in oligomeric and fibrillary Aβ (Frost and Li, 2017; Garwood et al., 2017). Additionally, the proinflammatory factors secreted by senescent astrocytes can also increase APP expression and Aβ generation in neurons (Del Bo et al., 1995). This means the higher release of SASP factors reduces the ability of the glia cells to promote Aβ clearance and facilitates its accumulation in the brain.

### Tau Accumulation and NFT Formation

Abnormal phosphorylation of the microtubule-associated protein tau and the subsequent accumulation of NFTs are the major pathological mechanisms of AD. Many studies have documented the vital role of senescent astrocytes in tau hyperphosphorylation and NFT formation (Bussian et al., 2018; Mendelsohn and Larrick, 2018). Musi et al. (2018) demonstrated that neurons carrying NFTs themselves become senescent, causing toxicity to nearby neurons. They performed a transcriptomic analysis of NFT-containing human neurons from the postmortem AD brain and revealed increased levels of the cellular senescence hallmark. The expression of senescence-associated genes, such as *p21*^*WAF1*^ and SASP proinflammatory genes, was significantly upregulated in the brain of the AD transgenic mouse model with NFTs. The authors treated with senolytics to remove the senescent cells, and found a reduction in total NFT density, neuronal loss, and ventricular enlargement. Although they link the expression of biomarkers of cellular senescence with the appearance of the NFTs in the AD mouse model, a causal relationship is not established, and the specific cell types involved cannot be identified. A recent study by Bussian et al. (2018) found the accumulation of p16^INK4A^-expression senescent astrocytes and microglia in the MAPT^P301S^PS19 mouse model of tau-dependent neurodegenerative disease. They then created a mouse strain (PS19/ATTAC) by crossing *INK-ATTAC* transgenic mice with the PS19 strain to remove p16-positive senescent cells through the administration of an inducer agent. They found that the clearance and removal of senescent astrocytes and microglial cells almost completely prevented hyperphosphorylated tau protein and NFT deposition in PS19/ATTAC mice, preserving cognitive function. Collectively, these results demonstrate that senescent astrocytes have a crucial role in tau accumulation and thus the etiopathology of tau-associated disease (Figure 2). These data also suggest a strong correlation between astrocyte senescence, Aβ formation, and tau accumulation, but the underlying mechanism tying together these characteristics with respect to senescent astrocytes in AD remains unclear and inconclusive.

### Synaptic Dysfunction and Neuronal Loss

Cognitive and memory impairment in AD is most likely caused by synapse loss and synapse dysfunction rather than mere neuronal loss or neuronal death (Terry et al., 1991; Hong et al., 2016). As the major glial cell types of the brain, astrocytes play a critical role in supporting neuronal growth and modulating synaptic function and transmission, yet the neuroprotective capacity of astrocytes decreased during aging (Pertusa et al., 2007; Yu et al., 2017). Although the mechanism of synapse dysfunction has not yet been fully elucidated, it is most likely a combination of Aβ plaque deposition, tau accumulation, and lesions (Miller et al., 2014; Piacentini et al., 2017). Astrocytes exhibit a senescence-like phenotype around Aβ plaques and NFTs in the brains of AD patients and AD mouse models (Bhat et al., 2012; Lok et al., 2013; Musi et al., 2018), and synapse dysfunction is also found mainly surrounding dense-core Aβ plaques (Koffie et al., 2009). These studies provide support for a relationship between senescent astrocytes and synaptic dysfunction or synapse loss in AD progression. *In vitro*, hippocampal neurons cocultured with senescent astrocytes showed suppressed synaptic maturation and transmission accompanied by a reduction in the size of synaptic vesicles (Kawano et al., 2012; Turnquist et al., 2016). The neuronal loss observed in AD can be attributed to the release of SASP factors such as IL-6. Furthermore, primary astrocytes from aged 5xFAD mice showed elevated SASP factor expression and neurotoxicity as well as impairment in supporting neuronal homeostasis (Iram et al., 2016). Moreover, senescent astrocytes secrete less neurotrophins such as nerve growth factor (NGF), insulin-like growth factor 1 (IGF-1), fibroblast growth factor 2 (FGF2), brain-derived neurotrophic factor (BDNF), and vascular endothelial growth factor (VEGF) (Bernal and Peterson, 2011; Turnquist et al., 2016; Bellaver et al., 2017), which may be lead to decreased neuronal growth and increased neuronal death in age-related neurological disorders.

Astrocytes also modulate synaptic properties via the abnormal release of the gliotransmitter GABA (Morel et al., 2017; Perez-Nievas and Serrano-Pozo, 2018). Moreover, senescent cortical astrocytes contribute to the impairment of synaptic plasticity and cognitive decline via a decline in the production of ATP, which is an important metabolic factor of neuronal activity (Lalo et al., 2014).

### Blood-Brain Barrier Dysfunction

The BBB limits the migration of cells and diffusion of molecules freely entering and exiting from the brain. The BBB is crucial for maintaining homeostasis of the brain microenvironment, which consists of perivascular microglia, endothelial cells, pericytes, neurons, and astrocytic end feet (Yamazaki and Kanekiyo, 2017). The impairment function of any of these cells could result in disturbance to the BBB.

In fact, the BBB is seen to leak both in normal aging and AD (Montagne et al., 2015; Yamazaki and Kanekiyo, 2017). However, the underlying mechanism of how aging disrupts the integrity of the BBB remains inconclusive. Astrocytes, although not involved in the formation of the BBB, participate in its maintenance and regulation. Senescent astrocytes produce a variety of SASP factors that influence the permeability of the BBB (Spampinato et al., 2017; Boisvert et al., 2018; Clarke et al., 2018). For instance, matrix metalloproteinases (MMPs), nitric oxide (NO), and VEGFs cause endothelial apoptosis and disrupt endothelial tight junctions (TJs) by downregulation of TJ-related proteins, resulting in BBB disruption (Abbott, 2002; Horng et al., 2017; Spampinato et al., 2017). Some of these molecules include transforming growth factor-β (TGFβ), glial cell–derived neurotrophic factor (GDNF), basic fibroblast growth factor (bFGF), IL-6 and upregulate endothelial cell adhesion molecules (CAMs), which induce leukocyte transmigration (Rochfort and Cummins, 2015). Interestingly, recent research has shown that the accumulation of senescent vascular cells results in compromised BBB integrity and reduced microvessel TJ coverage (Yamazaki et al., 2016). These senescent vascular smooth muscle cells (VSMCs) also contribute to the brain inflammation environment through the upregulation of proinflammatory cytokine IL-6 and chemokines, suggesting that senescent VSMCs have a crucial role in inducing age-dependent BBB breakdown. Yet, it is not clear whether the senescent astrocytes have a positive effect on VSMC senescence, and further studies should continue to explore the role of astrocyte senescence in increased BBB permeability during AD.

### Activation of Microglia and Promotion of Chronic Inflammation

Specifically, senescent astrocytes secrete SASP meditators such as IFNγ, CXCL10, IL-6, and TGFβ, which are capable of inducing inflammation (Bhat et al., 2012; Stamouli and Politis, 2016). For example, IFNγ is a potent regulatory cytokine that activates microglia and promotes inflammation in the AD brain (Blasko et al., 2004; Taylor et al., 2018). IL-6 is another typical SASP factor whose expression is upregulated in the aged brain and in those with AD. Its overexpression has been shown to drive neurodegeneration *in vitro* (Bhat et al., 2012). Several SASP factors, including IL-6, IL-1β, TNF-α, MMP-1, MMP-3, and MMP-10, have also been found to be elevated in the cerebrospinal fluid (CSF) and serum of AD patients (Wood et al., 1993; Blum-Degena et al., 1995; Leake et al., 2000; Horstmann et al., 2010; Gezen-Ak et al., 2013). This suggests that senescent astrocytes may sustain a proinflammatory status via the production of SASP factors and SASP-mediated microglia activation and inflammation may contribute to the pathogenesis of AD and aggravate the course of the disease.

## Astrocyte Senescence and Other Neurodegenerative Diseases

Senescent astrocytes are also more prominent in brain tissues from patients with other neurodegenerative diseases, such as Parkinson’s disease (PD) (Chinta et al., 2018; Scott and Williams-Gray, 2018) and amyotrophic lateral sclerosis (ALS) (Turnquist et al., 2016; Vazquez-Villasenor et al., 2019). PD is the second most common age-related neurodegenerative disease, characterized by loss of neurons in the substantia nigra pars compacta (SNpc), accumulation of α-synuclein, and presence of intraneuronal proteinaceous cytoplasmic inclusions (Lewy bodies) (Dauer and Przedborski, 2003). Increased levels of SASP-related factors, IL-1β, IL-6, and TNF-α, have been reported in the CSF, serum, and striatal dopaminergic regions of the patients with PD compared to controls (Mogi et al., 1994; Blum-Degena et al., 1995). Chinta et al. (2018) found that lamin B1 levels are decreased, while the expression of senescence-associated genes, *p16*^*INK4A*^ and *p21*^*WAF1*^, is increased in astrocytes of SNpc tissues in PD patients. Additionally, conditioned media from senescent astrocytes significantly reduced the viability of dopaminergic neurons and the proliferation and migration of neural progenitor cells (NPCs). Most importantly, they found that selective elimination of senescent astrocytes could repress the development of paraquat-induced neurodegenerative phenotypes associated with sporadic PD (Chinta et al., 2018), demonstrating that astrocyte senescence might be a critical mechanism for PD neurodegeneration. ALS is a fatal neurodegenerative disease characterized by the loss of upper and lower motor neurons (MNs). Postmortem brain tissue from ALS patients exhibits increased numbers of senescent astrocytes (Turnquist et al., 2016). This is further supported by animal models of ALS that show increased expression of the senescence marker p16^INK4A^ in GFAP-positive astrocytes within lumbar spinal cord sections that typically surround damaged motor neurons (Trias et al., 2019). Moreover, SASP cytokine *IL-6* as well as p16^INK4A^ and *p21*^*WAF1*^ were more remarkably upregulated in ALS compared to AD (Turnquist et al., 2016). Aged astrocytes are less supportive to motor neuron function (Das and Svendsen, 2015; Turnquist et al., 2016). Senescent astrocytes might exert their neurotoxic effect on neurons through the release of SASP factors, exacerbating neuroinflammation. However, it is not clear whether prevention or clearance of senescent cells in ALS could delay disease progression as is seen in PD models.

## Therapeutic Applications

Currently, most of the therapeutic approaches of AD focus mainly on the modulation of Aβ production by inhibiting Aβ generation and enhancing Aβ degradation or reducing tau protein deposits and NFT accumulation (Citron, 2010). Others attempt at targeting apoE, neuroinflammation, metabolic dysfunction, and epigenetic changes (Long and Holtzman, 2019). Despite the fact that there are more than 100 different compounds in various stages of clinical trials being tested for use in early-, mid- or late-stage AD, there are few efficacious therapeutic options available (Hara et al., 2019; Long and Holtzman, 2019). The failure of these trials emphasizes a great need for different pharmaceutical therapies to prevent or delay the progression of AD.

Aging is a critical risk factor for most age-related neurodegenerative disease, including AD (Baker and Petersen, 2018; Kritsilis et al., 2018; Hara et al., 2019). A hallmark of aging is senescent cell accumulation. In recent years, a significant finding in aging and age-related diseases research is that selective elimination of senescent cells can extend lifespan and slow the progression of diseases *in vivo* without triggering negative side effects (Baker et al., 2011, 2016; Jeon et al., 2017). This strategy is referred to as senolysis. As mentioned earlier, a great number of studies indicated that astrocyte senescence plays a crucial role in the pathogenesis of AD. Senolytic therapeutic strategies that safely and effectively reduce the detrimental effects of senescent astrocytes, such as the neutralization of SASP or targeted clearance of senescent astrocytes, are gaining considerable attention in AD.

### Alleviate Astrocyte Senescence and Decrease SASP Level

Several lines of research suggest that astrocyte senescence occurs in the early stage of disease progression and may induce or aggravate other neurodegenerative pathologies. Thus, therapies to alleviate astrocyte senescence could prevent the onset of AD or delay its progress. In AD, senescent astrocytes exert deleterious effects on neurons via both reduced secretion of neurotrophic growth factors (NGF, IGF-1) and elevated production of SASP factors (Turnquist et al., 2016). Turnquist et al. (2016, 2019) demonstrated that overexpression of Δ133p53 or downregulation of *p53*β can alleviate astrocyte senescence, repress SASP production, and consequently prevent neuronal apoptosis and loss. Additionally, in astrocytes, Δ133p53-mediated upregulation of neurotrophic factors induced a neuroprotection effect. This shift secretion profile demonstrated that SASP may be a promising therapeutic method for slowing the progression of AD. There are two more commonly used SASP neutralization therapies: inhibiting SASP-initiated signaling and blocking the activity of particular components of the SASP.

As mentioned earlier, astrocytic SASP genes are upregulated in a p38/MAPK and NF-κB signaling-dependent manner. Thus, targeting the NF-κB pathway decreased the production of SASP. Similarly, pharmacological inhibition of p38/MAPK abolished SASP secretion by senescent astrocytes. Yet, not all SASP factors are altered in senescent astrocytes. Because several SASP factors are essential to maintaining cell senescence, inhibition of pro-SASP signaling can increase the risk of cancer development (Coppe et al., 2010; Hoare et al., 2016). Alternatively, blockade of specific SASP factors, such as IL-6, is also a viable strategy. Specifically, using IL-6 monoclonal neutralizing antibodies, which are already approved for clinical applications, i.e., siltuximab (anti-human IL-6) or tocilizumab (anti-human IL-6R), could show some promise.

### Clearance of Senescent Astrocytes (Senotherapy)

In many age-related disorders such as osteoarthritis, atherosclerosis, and diabetes mellitus type 2, the removal of senescent cells of transgenic mice models has shown an impaired associated pathology and extended the healthy lifespan (Baker et al., 2011, 2016; Jeon et al., 2017). Success has also been observed in a mouse model of tau-associated pathogenesis. This study was the first to demonstrate a causal relationship between glial senescence and neurodegeneration (Bussian et al., 2018). In this study, Bussian et al. found accumulations of senescent astrocytes and microglia in tau-associated neurodegenerative disease model mice. Elimination of these senescent cells via a genetic approach can reduce tau deposition and prevent the degeneration of cortical and hippocampal neurons. Besides, a new set of pharmacological drugs has been proven to show a similar effect, termed senolytics, which include ABT263, quercetin, and dasatinib (Zhu et al., 2014). Indeed, these pharmacological agents have already been successful in preclinical and clinical trials in several age-associated diseases (reviewed by Childs et al., 2017). Most recently, Zhang X. et al. (2019) showed that clearance of senescent oligodendrocyte progenitor cells in AD model mice with senolytic agents could lessen the Aβ plaque load, reduce neuroinflammation, and ameliorate cognitive deficits. This seno-therapeutic approach is currently being tested in neurodegenerative diseases and despite expected challenges and difficulties, more detailed investigation is warranted.

## Conclusion and Outlook

Astrocytes undergo degeneration and senescence in the early stages of AD progression, which may alter the microenvironment of the brain and contribute to early cognitive deficits. However, how senescent astrocytes actively and exactly contribute to the progression of AD is still to be fully characterized. Fortunately, targeting astrocyte senescence using a senolytics approach, among others, is beginning to emerge in AD treatments, with evidence in AD animal models already showing promise. Additionally, other cell types in the brain, including microglia (Flanary, 2005), oligodendrocytes (Zhang P. et al., 2019), neural stem cells (He et al., 2013), and neurons (Jurk et al., 2012), showed senescent phenotypes that are also involved in development of AD. Therefore, the link between cellular senescence of other CNS cell types and AD needs to be further explored.

Furthermore, extensive astrocyte senescence has also been found in other age-related neurodegenerative diseases such as PD and ALS. This indicates that astrocyte senescence seems to be a common characteristic of neurodegeneration, and future research needs to examine this phenomenon in more detail. Additionally, the pathologies of neurodegenerative diseases induced by senescent astrocytes need to be further understood. Such future studies could increase our knowledge of cellular senescence and neurodegeneration medicine.

## Author Contributions

XH conceived and wrote the manuscript. TZ and HL contributed to manuscript preparation. YM and XG designed the concept of this study and discussed the results with all authors.

## Conflict of Interest

The authors declare that the research was conducted in the absence of any commercial or financial relationships that could be construed as a potential conflict of interest.
